# Supplementary material for: Amphotericin B promotes respiratory viral entry by enhancing late endosomal maturation and fusion via glucocerebrosidase-mediated ceramide remodeling
Source: Nat Commun. 2026 Mar 9;17:3670. doi: 10.1038/s41467-026-70095-x (PMC13100133; doi:10.1038/s41467-026-70095-x)
Supplement: Supplementary file 1 — Supplementary Information [file 41467_2026_70095_MOESM1_ESM.pdf]

Figure S1

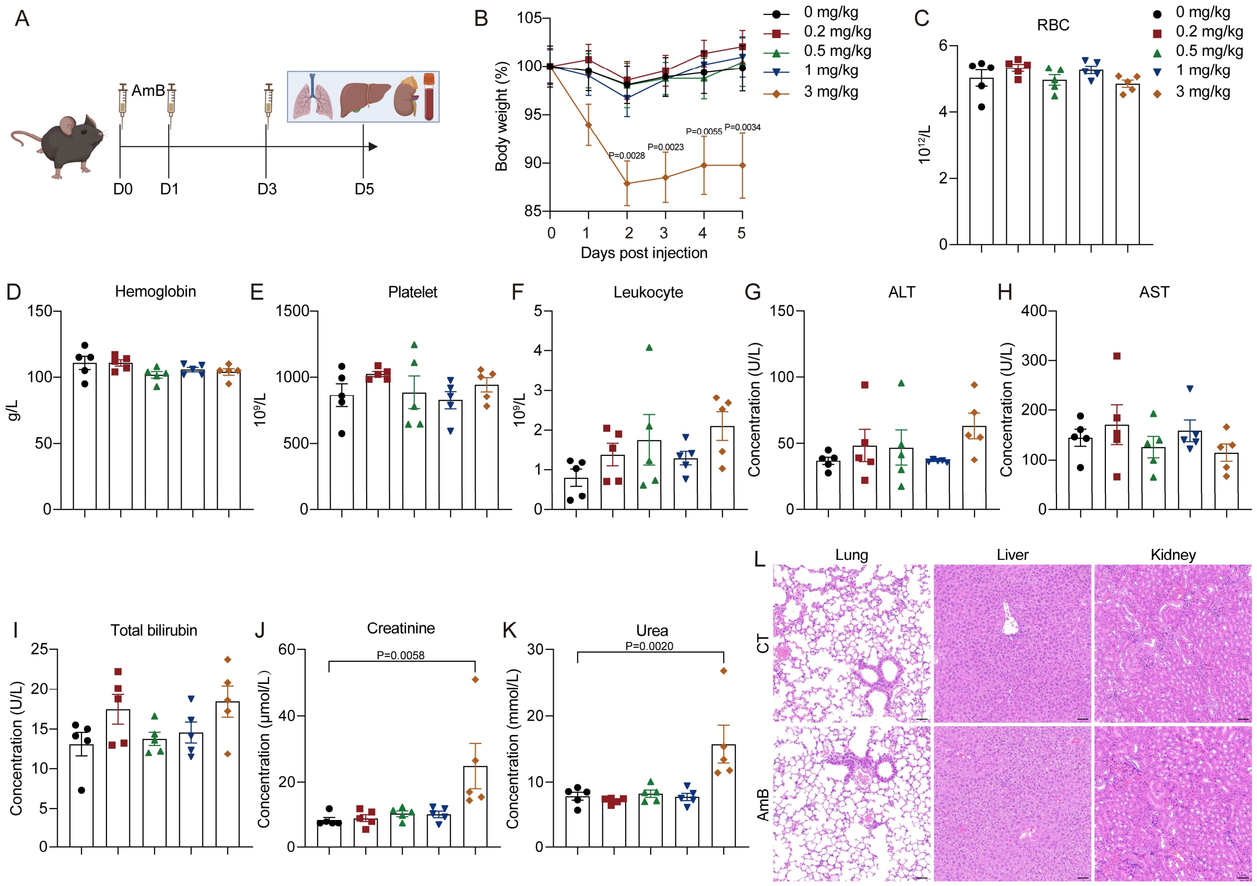

Fig. S1. Dose-dependent toxicity evaluation of amphotericin B (AmB) in murine models.

(A) Schematic illustration of experimental design. Mice received intraperitoneal injections of AmB at doses of 0, 0.2, 0.5, 1, or 3 mg/kg at 0, 24, and 72 h. On day 5, blood and tissue samples (lung, liver, and kidney) were collected for multimodal analyses. Created in BioRender. Li, S. (2026) https://BioRender.com/dljer7j.

(B) Longitudinal body weight monitoring throughout the study.

(C–F) Peripheral blood analyses were performed on day 5 to assess red blood cell (RBC) count (C), hemoglobin concentration (D), platelet count (E), and leukocyte count (F).

(G–K) Liver function was determined by measuring alanine aminotransferase (ALT, G), aspartate aminotransferase (AST, H), and total bilirubin (I) at 5 days post injection, while kidney function was assessed based on creatinine (J) and urea (K) levels.

(L) Representative H&E-stained sections of lung, liver, and kidney from mice treated with the high dose (3 mg/kg) of AmB compared to controls (CT). Scale bars, 50 μm.

Data are expressed as mean ± SEM. n = 5 mice per group (B–L). Statistical analysis was performed using two-way ANOVA (B) and one-way ANOVA (C–K) followed by Dunnett’s multiple comparisons test. Source data are provided as a Source Data file.

Figure S2

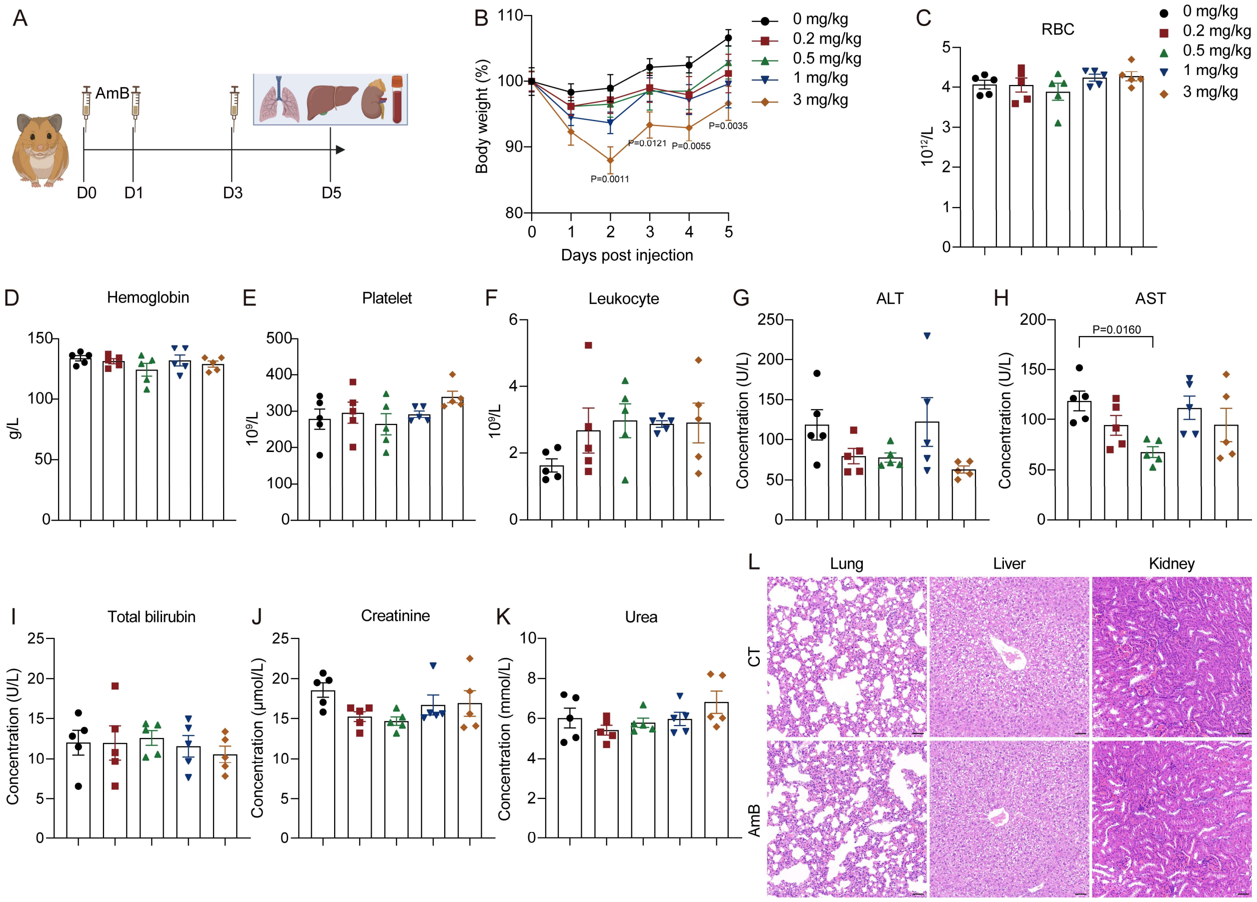

Fig. S2. Toxic responses to graded amphotericin B (AmB) doses in hamsters.

(A) Schematic illustration of experimental design. Hamsters received intraperitoneal injections of AmB at doses of 0, 0.2, 0.5, 1, or 3 mg/kg at 0, 24, and 72 h. On day 5, blood and tissue samples (lung, liver, and kidney) were collected for multimodal analyses. Created in BioRender. Li, S. (2026) https://BioRender.com/gjb7vn2.

(B) Dynamics of body weight during the study.

(C–F) Hematological evaluations in hamsters on day 5, including red blood cell (RBC) count (C), hemoglobin concentration (D), platelet count (E), and leukocyte count (F).

(G–K) Serum biochemical analyses performed on day 5 included measurements of liver function markers alanine aminotransferase (ALT, G), aspartate aminotransferase (AST, H), and total bilirubin (I), as well as kidney function markers creatinine (J) and urea (K).

(L) Representative H&E-stained sections of the lung, liver, and kidney from hamsters receiving the high dose (3 mg/kg) of AmB are presented alongside those from control animals (CT). Scale bars, 50 µm.

Data are expressed as mean ± SEM. n = 5 hamsters per group (B–L). Statistical analysis was performed using two-way ANOVA (B) and one-way ANOVA (C–K) followed by Dunnett's multiple comparisons test. Source data are provided as a Source Data file.

Figure S3

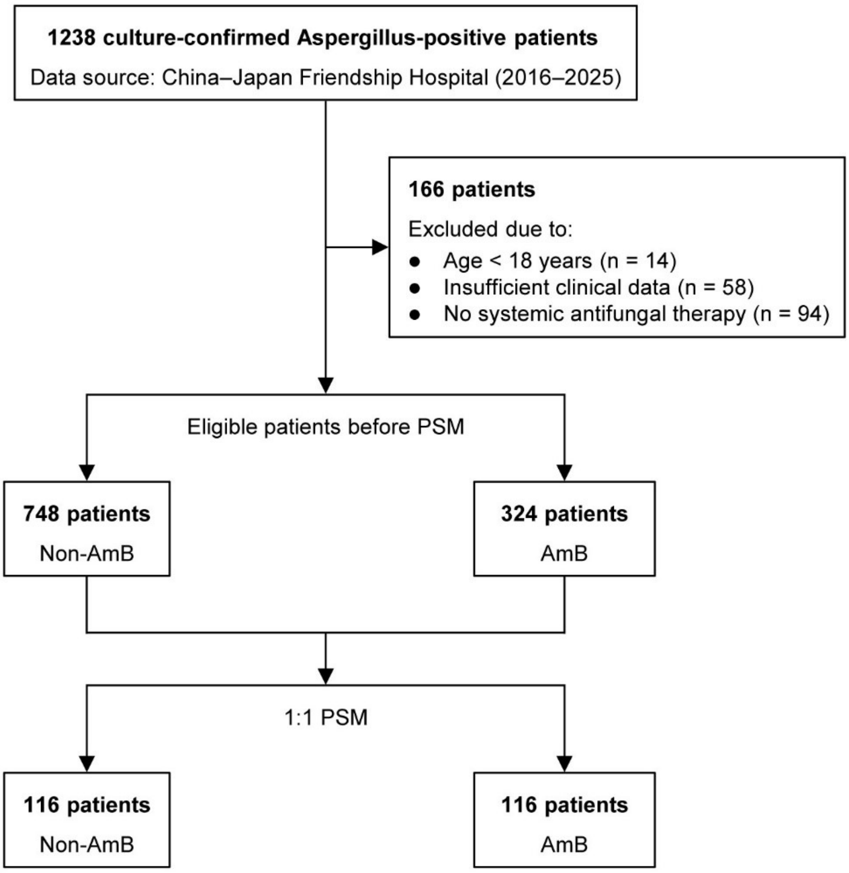

Fig. S3. Flow diagram of included patients and propensity score matching (PSM).

Figure S4

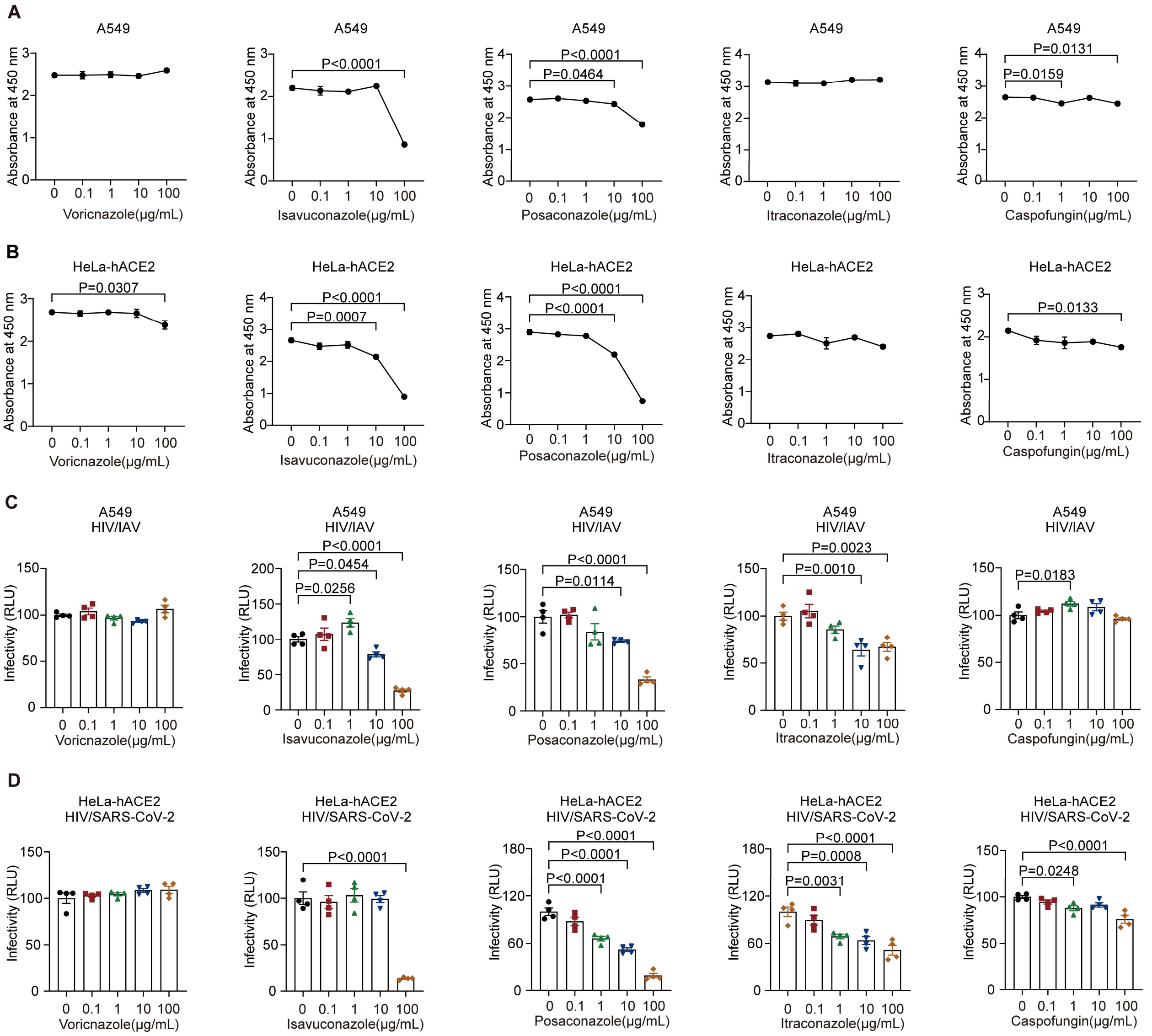

Fig. S4. Cytotoxicity of antifungal drugs and their effect on pseudoviral entry.

(A–B) Cell viability of A549 (A) and HeLa-hACE2 (B) cells treated with voriconazole, isavuconazole, posaconazole, itraconazole, or caspofungin for 48 h, assessed by Cell Counting Kit-8 (CCK-8) assay.

(C–D) Pseudoviral entry efficiency in A549 (C) and HeLa-hACE2 (D) cells. Cells were pretreated with these compounds for 1 h, then infected with HIV/IAV (C) or HIV/SARS-CoV-2 (D) pseudoviruses. Luciferase activity was measured at 48 h post-infection.

Data are presented as mean ± SEM. n = 4 independent experiments (A–D). Statistical analysis was performed using one-way ANOVA followed by Dunnett's multiple comparisons test (A–D). Source data are provided as a Source Data file.

Figure S5

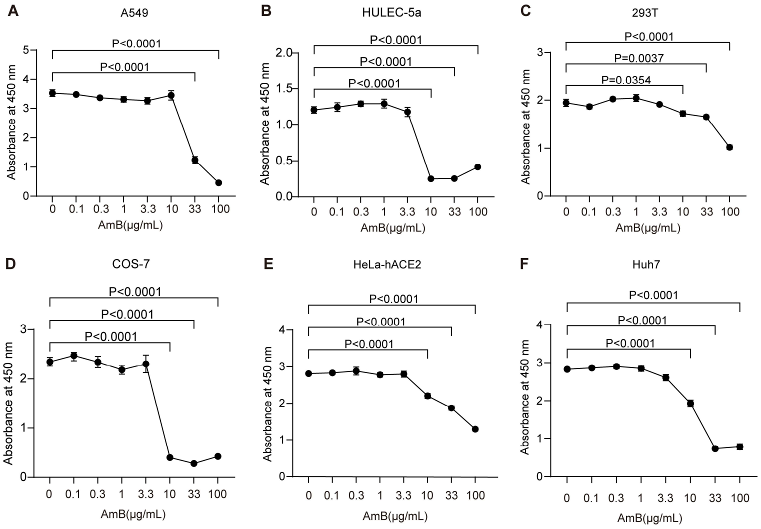

Fig. S5. Evaluation of amphotericin B (AmB)-induced cytotoxicity in various cell lines using the Cell Counting Kit-8 (CCK-8) assay.

(A–F) A549 (A), HULEC-5a (B), 293T (C), COS-7 (D), HeLa-hACE2 (E), and Huh7 (F) cells were exposed to increasing concentrations of AmB for 48 h, and cell viability was determined by measuring the absorbance at 450 nm.

Data are expressed as mean ± SEM. n = 4 independent experiments (A–F). Statistical analysis was performed using one-way ANOVA followed by Dunnett's multiple comparisons test (A–F). Source data are provided as a Source Data file.

Figure S6

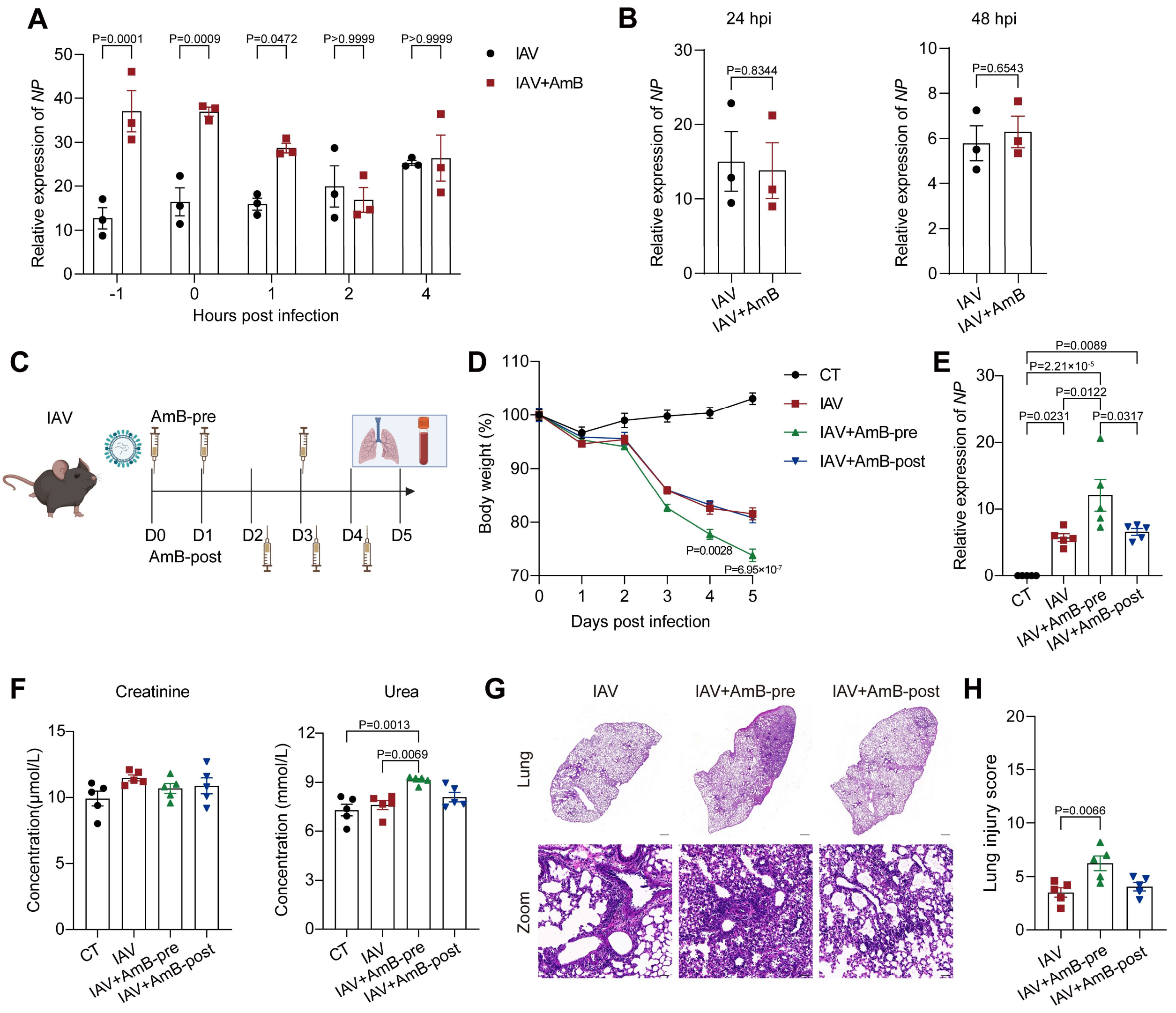

Fig. S6. Amphotericin B (AmB) enhances viral infection only when administered during the early phase.

(A) In the continuous presence of 50 μg/mL cycloheximide, A549 cells were infected with influenza A virus (H1N1) strain A/PR/8/34 (IAV; MOI = 0.1) and treated with 1 μg/mL AmB at the indicated times relative to infection (−1, 0, +1, +2, or +4 h). *Nucleoprotein (NP)* RNA levels were measured by RT-qPCR at 6 h post-infection (hpi).

(B) Following IAV infection (MOI = 0.1), A549 cells were treated with 1 μg/mL AmB at 24 or 48 hpi, and *NP* RNA levels were quantified by RT-qPCR 24 h later.

(C) Mice were inoculated intranasally with IAV (200 PFU). In the pre-administration group (IAV+AmB-pre), 1 mg/kg AmB was administered intraperitoneally 1 h before infection and again at 1 and 3 days post-infection (dpi). In the post-administration group (IAV+AmB-post), AmB was first given at 2 dpi and repeated at 3 and 4 dpi. Control groups included infected only (IAV) and uninfected (CT) mice. Lungs and serum were collected at 5 dpi. Created in BioRender. Li, S. (2026) https://BioRender.com/7u40m2d.

(D) Body weight change of mice over time. Statistical significance is indicated for IAV+AmB-pre compared to IAV.

(E) Viral *NP* RNA levels in lung homogenates at 5 dpi were measured using RT-qPCR.

(F) Renal function assessed by serum creatinine and urea levels at 5 dpi.

(G–H) Representative hematoxylin and eosin (H&E)–stained lung sections are shown (G), along with pathological scoring for inflammation, hemorrhage, edema, atelectasis, and necrosis (H). Scale bars: 0.5 mm (grey), 50 μm (black).

Data are presented as mean ± SEM. n = 3 independent experiments (A–B); n = 5 mice per group (D–H). Statistical analysis was performed using two-way ANOVA followed by Bonferroni's (A) or Tukey's (D) multiple comparisons tests, two-tailed unpaired Student's t-test (B), and one-way ANOVA followed by Tukey's (E–F) or Dunnett's (H) multiple comparisons tests. Source data are provided as a Source Data file.

Figure S7

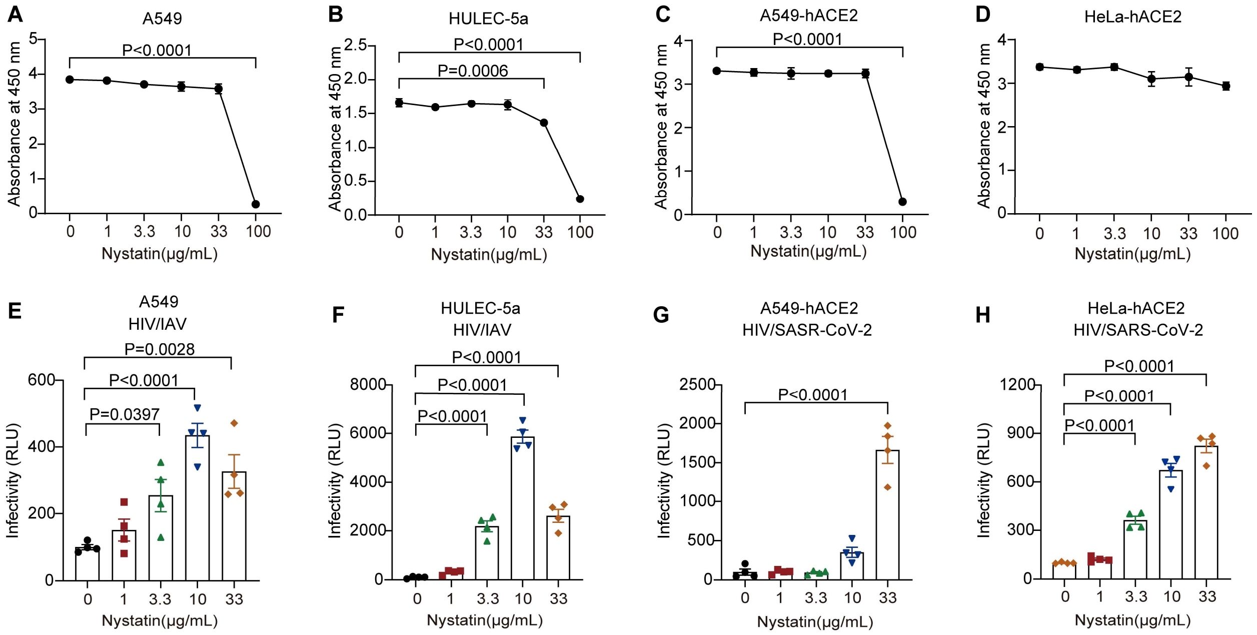

Fig. S7. Cytotoxicity profiling of nystatin and its enhancement on pseudoviral entry.

(A–D) Cell viability of A549 (A), HULEC-5a (B), A549-hACE2 (C), and HeLa-hACE2 (D) cells treated with nystatin for 48 h was assessed by Cell Counting Kit-8 (CCK-8) assay.

(E–H) A549 (E) and HULEC-5a (F) cells were infected with HIV/IAV pseudoviruses, while A549-hACE2 (G) and HeLa-hACE2 (H) cells were challenged with HIV/SARS-CoV-2 pseudoviruses. Viral entry efficiency was evaluated by measuring luciferase activity 48 h post-infection following a 1-h pretreatment with nystatin.

Data are expressed as mean ± SEM. n = 4 independent experiments (A–H). Statistical analysis was performed using one-way ANOVA followed by Dunnett's multiple comparisons test (A–H). Source data are provided as a Source Data file.

Figure S8

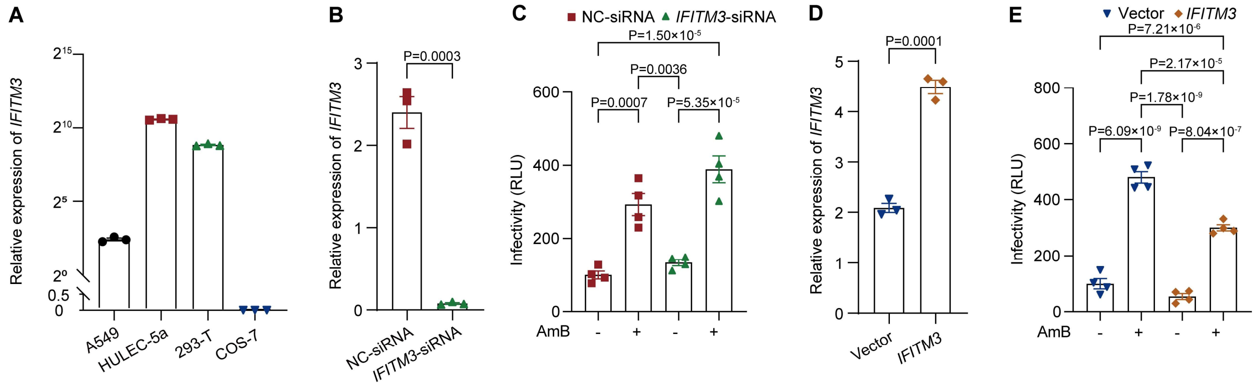

Fig. S8. Amphotericin B (AmB) enhances viral entry independent of interferon-induced transmembrane protein 3 (IFITM3)-mediated restriction.

(A) Basal *IFITM3* mRNA levels in A549, HULEC-5a, 293T, and COS-7 cells were determined by RT-qPCR.

(B) Validation of *IFITM3* knockdown in A549 cells by RT-qPCR 48 h post-transfection with either a negative control siRNA (NC-siRNA) or an *IFITM3*-targeting siRNA (*IFITM3*-siRNA).

(C) After transfection with siRNAs, A549 cells were pretreated with 1 µg/mL AmB for 1 h and then infected with HIV/IAV pseudoviruses. Viral entry was quantified by measuring luciferase activity 48 h post-infection (hpi).

(D) Validation of plasmid-based transient *IFITM3* overexpression in A549 cells by RT-qPCR 48 h after transfection.

(E) Following transfection with either an empty vector or *IFITM3*-expressing plasmids, cells were pretreated with AmB and then infected with HIV/IAV pseudoviruses. Viral entry was quantified based on luciferase activity at 48 hpi.

Data are presented as mean ± SEM. n = 3 independent experiments (A–B, D); n = 4 independent experiments (C, E). Statistical analysis was performed using two-tailed unpaired Student's t-test (B, D), and one-way ANOVA followed by Tukey's multiple comparisons tests (C, E). Source data are provided as a Source Data file.

Figure S9

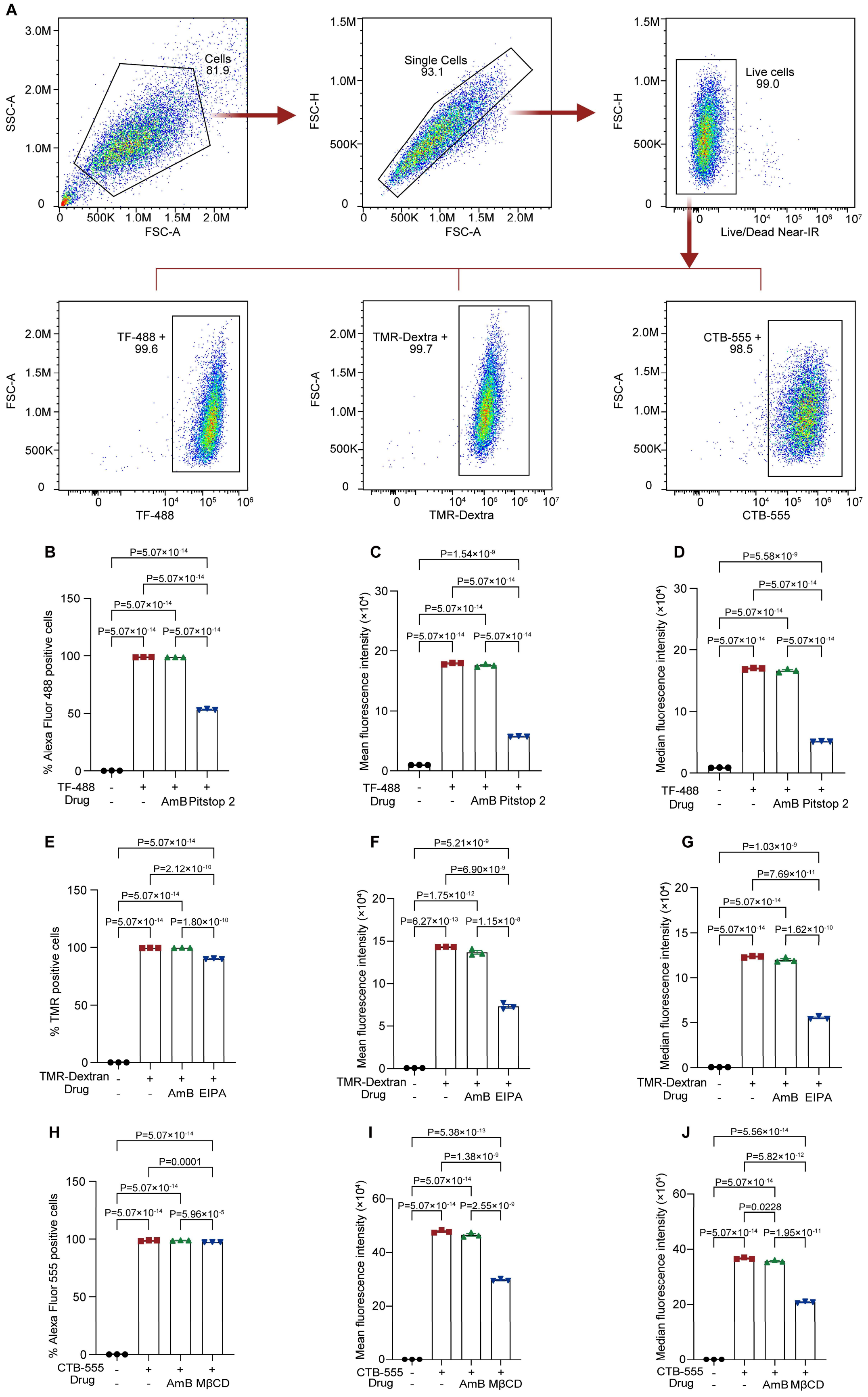
A
Cells
81.9
SSC-A
FSC-A
Single Cells
93.1
FSC-H
Live cells
99.0
Live/Dead Near-IR
TF-488 +
99.6
FSC-A
TF-488
TMR-Dextra +
99.7
TMR-Dextra
CTB-555 +
98.5
CTB-555
B
% Alexa Fluor 488 positive cells
P=5.07×10⁻¹⁴
TF-488 - + + +
Drug - - AmB Pitstop 2
C
Mean fluorescence intensity (×10⁴)
P=1.54×10⁻⁹
P=5.07×10⁻¹⁴
D
Median fluorescence intensity (×10⁴)
P=5.58×10⁻⁹
P=5.07×10⁻¹⁴
E
% TMR positive cells
P=5.07×10⁻¹⁴
P=2.12×10⁻¹⁰
P=1.80×10⁻¹⁰
TMR-Dextran - + + +
Drug - - AmB EIPA
F
P=5.21×10⁻⁹
P=6.90×10⁻⁹
P=1.75×10⁻¹²
P=6.27×10⁻¹³
P=1.15×10⁻⁸
G
P=1.03×10⁻⁹
P=7.69×10⁻¹¹
P=5.07×10⁻¹⁴
P=1.62×10⁻¹⁰
H
% Alexa Fluor 555 positive cells
P=5.07×10⁻¹⁴
P=0.0001
P=5.96×10⁻⁵
CTB-555 - + + +
Drug - - AmB MβCD
I
P=5.38×10⁻¹³
P=1.38×10⁻⁹
P=5.07×10⁻¹⁴
P=2.55×10⁻⁹
J
P=5.56×10⁻¹⁴
P=5.82×10⁻¹²
P=5.07×10⁻¹⁴
P=0.0228
P=1.95×10⁻¹¹

Fig. S9. Amphotericin B (AmB) does not affect clathrin-mediated endocytosis, macropinocytosis, or caveolin-dependent uptake.

(A) Representative gating strategy for flow cytometry analysis. Cells were initially identified based on forward scatter (FSC) and side scatter (SSC), followed by doublet exclusion and viability staining using a Live/Dead Near-Infrared (Near-IR) stain. Specific positive gates were then established to identify cells that had internalized Alexa Fluor 488-conjugated transferrin (TF-488), tetramethylrhodamine-labeled 70-kDa dextran (TMR-Dextran), or Alexa Fluor 555-conjugated cholera toxin subunit B (CTB-555).

(B–D) A549 cells were pretreated with 1 μg/mL AmB or 20 μM Pitstop 2 for 2 h, then incubated with 25 μg/mL TF-488 for 5 min at 37 °C. TF-488 internalization was assessed by flow cytometry, showing the percentage of Alexa Fluor 488-positive cells (B), mean (C) and median fluorescence intensity (D).

(E–G) A549 cells were pretreated with 1 μg/mL AmB or 50 μM EIPA for 2 h, followed by a 40-min exposure to 1 mg/mL TMR-Dextran at 37 °C. Flow cytometry was used to quantify the percentage of TMR-positive cells (E), mean (F) and median fluorescence intensity (G).

(H–J) Following pretreatment with 1 μg/mL AmB or 5 mM methyl-β-cyclodextrin (MβCD) for 2 h, A549 cells were incubated with 10 μg/mL CTB-555 for 30 min at 37 °C. Uptake of CTB-555 was analyzed by flow cytometry, presenting the percentage of Alexa Fluor 555-positive cells (H), mean (I) and median fluorescence intensity (J).

Data are presented as mean ± SEM. n = 3 independent experiments (B–J). Statistical analysis was performed using one-way ANOVA followed by Tukey's multiple comparisons test (B–J). Source data are provided as a Source Data file.

Figure S10

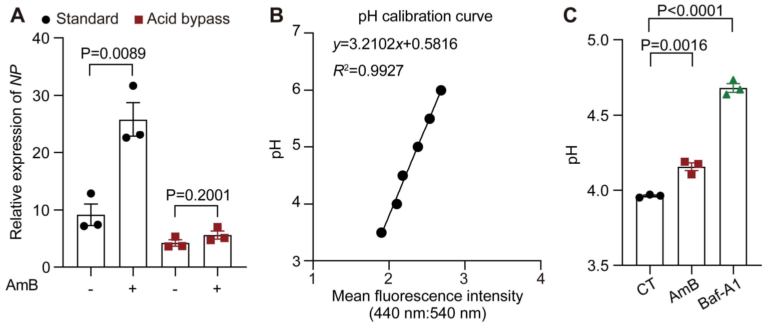

Fig. S10. Amphotericin B (AmB) does not promote virus fusion at the plasma membrane and has a minimal effect on endosomal pH.

(A) All procedures were performed in the continuous presence of 50 µg/mL cycloheximide. A549 cells were pretreated with 1 µg/mL AmB for 1 h, incubated with influenza A virus (H1N1) strain A/PR/8/34 (MOI = 0.1) at 4 °C for 1 h, and then pulsed for 2 min at 37 °C in either standard infection medium or acid-bypass fusion medium. Cells were further cultured for 5 h in infection medium containing 10 mM NH_4Cl before viral *nucleoprotein (NP)* RNA levels were quantified by RT-qPCR.

(B) A549 cells loaded with LysoSensor™ were equilibrated in calibration buffers ranging from pH 3.5 to 6.0, and the calibration curve was constructed.

(C) A549 cells were treated with 1 µg/mL AmB or 20 nM bafilomycin A1 (Baf-A1) for 2 h and subsequently loaded with 1 µM LysoSensor™ for 5 min at 37 °C. The pH of acidic organelles was determined using the calibration curve established in panel B.

Data are presented as mean ± SEM. n = 3 independent experiments (A–C). Statistical analysis was performed using two-tailed unpaired Student's t-test (A), and one-way ANOVA followed by Dunnett's multiple comparisons test (C). Source data are provided as a Source Data file.

Figure S11

A

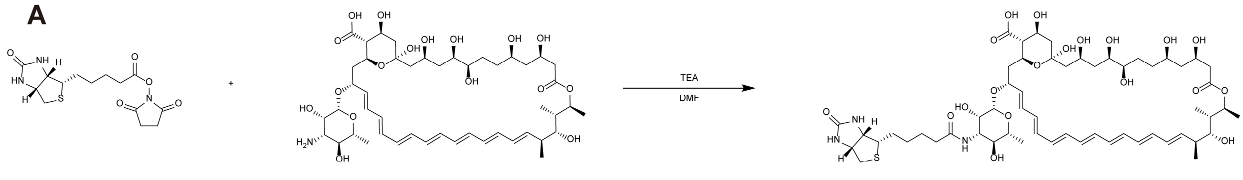

B

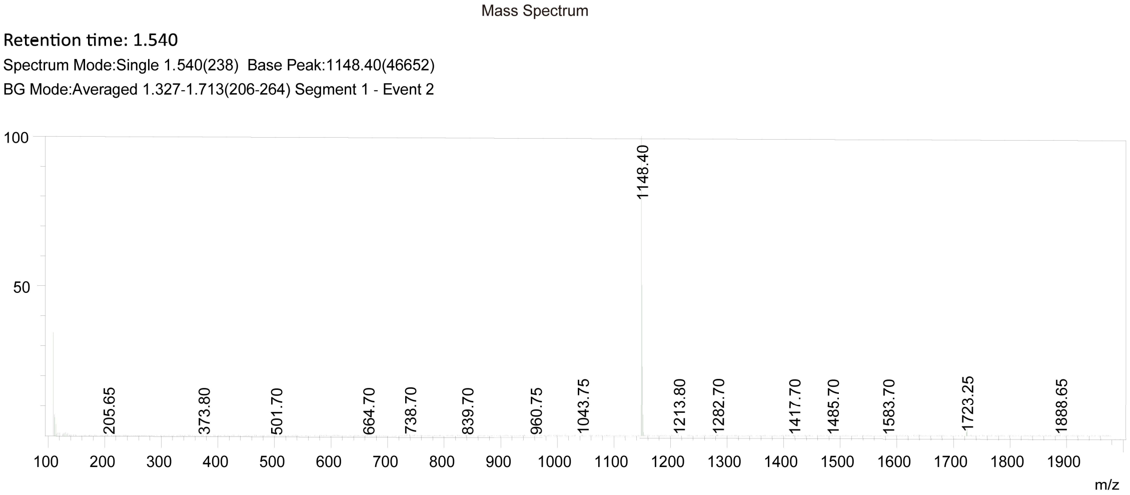

Fig. S11. Synthesis and characterization of the amphotericin B (AmB)-biotin conjugate.

(A) Schematic representation of the conjugation reaction between AmB and N-hydroxysuccinimide-biotin. The reaction was performed in dimethylformamide (DMF) containing triethylamine (TEA).

(B) Characterization of the AmB-biotin conjugate by mass spectrometry.

Figure S12

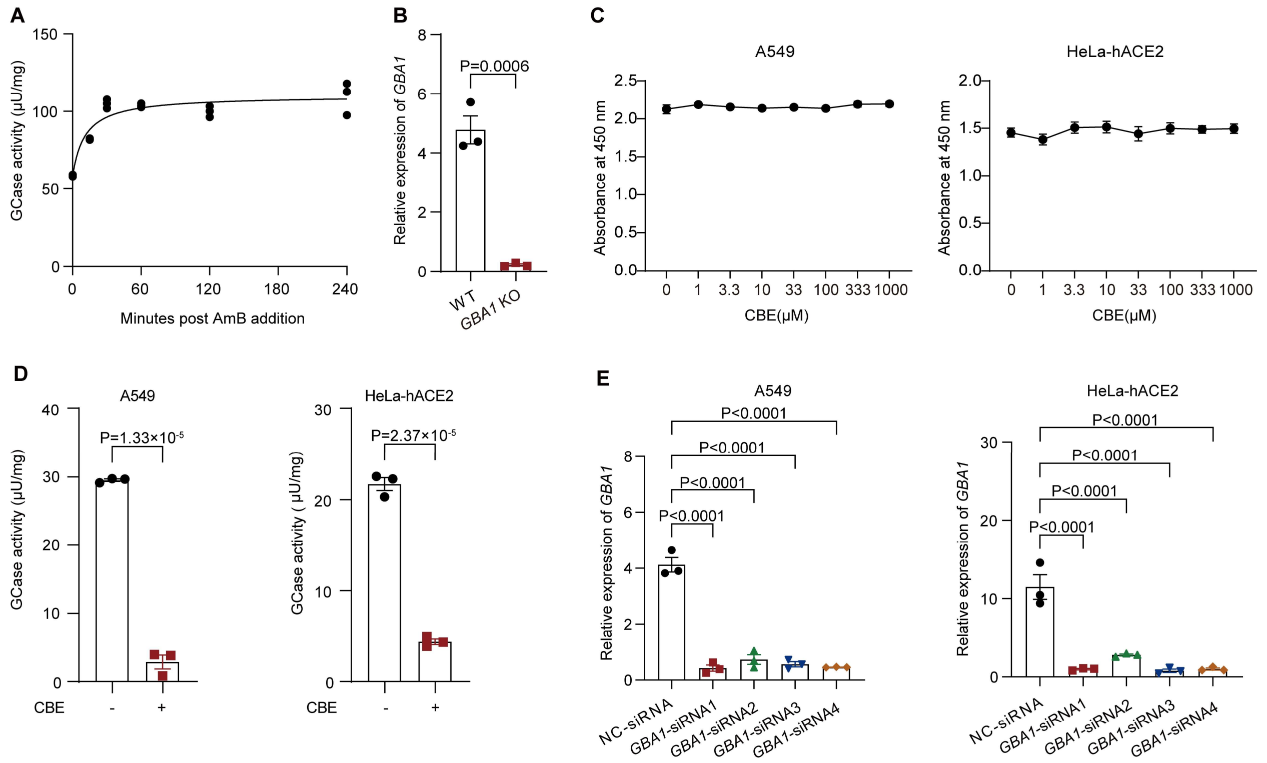

Fig. S12. Amphotericin B (AmB) rapidly activates glucocerebrosidase (GCase), and characterization of conduritol B epoxide (CBE) cytotoxicity and the efficiency of *GBA1* genetic perturbation.

(A) GCase activity was measured in A549 cell lysates after treatment with 1 μg/mL AmB for the indicated times using a fluorogenic substrate assay.

(B) Validation of *GBA1* knockout (KO) through RT-qPCR.

(C) A549 and HeLa-hACE2 cells were treated with increasing concentrations of CBE for 48 h, and cell viability was determined by Cell Counting Kit-8 (CCK-8) assay.

(D) GCase activity was quantified in cell lysates from A549 and HeLa-hACE2 cells pretreated with 100 μM CBE for 4 h.

(E) RT-qPCR validation of *GBA1* knockdown efficiency in A549 and HeLa-hACE2 cells.

Data are presented as mean ± SEM. n = 3 independent experiments (A–B, D–E); n = 4 independent experiments (C). Statistical analysis was performed using two-tailed unpaired Student's t-test (B, D), and one-way ANOVA followed by Dunnett's multiple comparisons test (C, E). Source data are provided as a Source Data file.

Figure S13

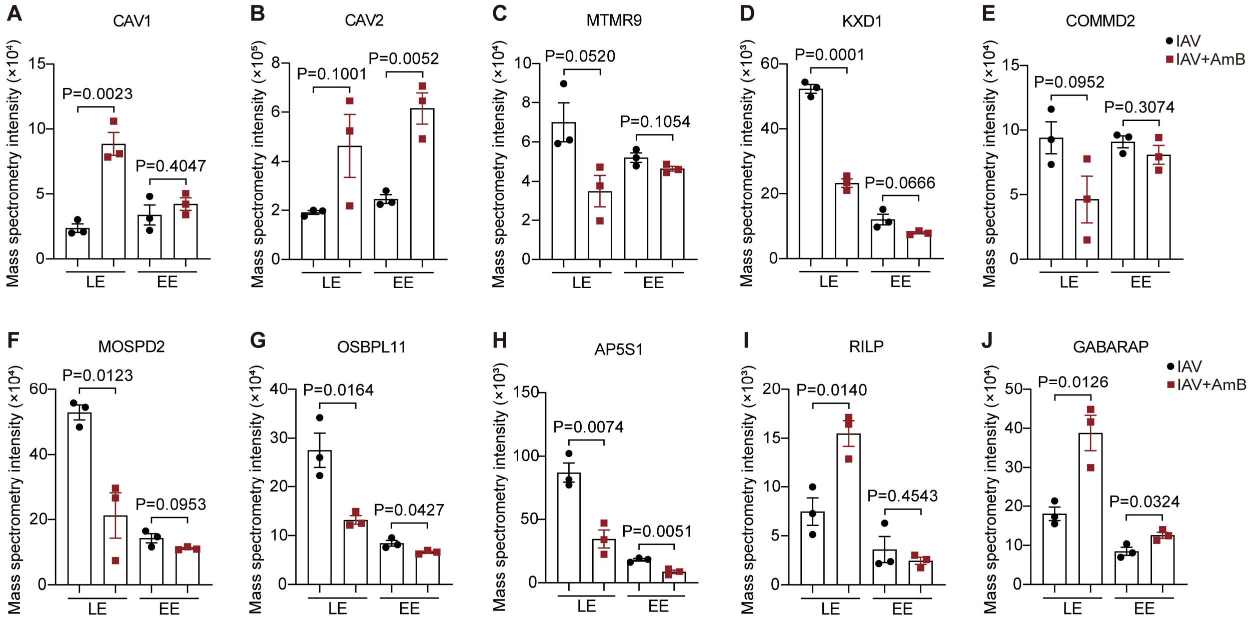

Fig. S13. Amphotericin B (AmB) remodels the endosomal proteome.

(A–J) A549 cells were pretreated with 1 μg/mL AmB or vehicle for 1 h, infected with influenza A virus (H1N1) strain A/PR/8/34 (MOI = 1) for 4 h, and then fractionated to isolate early and late endosomes for analysis by data-independent acquisition-based quantitative proteomics. The relative abundances of the indicated endosomal proteins are shown: CAV1 (A), CAV2 (B), MTMR9 (C), KXD1 (D), COMMD2 (E), MOSPD2 (F), OSBPL11 (G), AP5S1 (H), RILP (I), and GABARAP (J).

Data are presented as mean ± SEM. n = 3 independent experiments (A–J). Statistical analysis was performed using two-tailed unpaired Student's t-test (A–J). Source data are provided as a Source Data file.

Table S1. Annual counts and proportions of patients treated with amphotericin B (AmB) among those with culture-confirmed *Aspergillus* infections requiring antifungal therapy (2016–2025)

Year	Total (n = 1072)	Non-AmB (n = 748)	AmB (n = 324)
2016	11	9 (81.82)	2 (18.18)
2017	59	46 (77.97)	13 (22.03)
2018	40	31 (77.50)	9 (22.50)
2019	62	37 (59.68)	25 (40.32)
2020	47	33 (70.21)	14 (29.79)
2021	112	65 (58.04)	47 (41.96)
2022	138	91 (65.94)	47 (34.06)
2023	277	199 (71.84)	78 (28.16)
2024	216	158 (73.15)	58 (26.85)
2025	110	79 (71.82)	31 (28.18)

Data are presented as n (%) per year.

Table S2. Characteristics of patients with *Aspergillus* infection according to amphotericin B (AmB) exposure, before and after propensity score matching (PSM)

Variable	Before PSM				After PSM			
	Total (n = 1072)	Non-AmB (n = 748)	AmB (n = 324)	P	Total (n = 232)	Non-AmB (n = 116)	AmB (n = 116)	P
**Demographics**								
Age, years	64.00 (55.00, 71.00)	65.00 (57.00, 73.00)	61.00 (52.00, 68.00)	**1.479×10^{-9}**	63.00 (52.75, 70.00)	64.00 (51.75, 72.00)	63.00 (54.00, 69.00)	0.714
Male sex	716 (66.79)	478 (63.90)	238 (73.46)	**0.002**	160 (68.97)	79 (68.10)	81 (69.83)	0.777
BMI, kg/m^2	22.49 (19.92, 25.35)	22.58 (19.95, 25.32)	22.41 (19.90, 25.40)	0.744	22.38 (19.59, 24.98)	22.89 (19.81, 25.13)	22.12 (19.46, 24.65)	0.243
**Vital signs**								
Temperature, °C	36.50 (36.20, 36.80)	36.50 (36.20, 36.80)	36.50 (36.20, 36.80)	0.948	36.50 (36.20, 36.80)	36.50 (36.20, 36.80)	36.50 (36.30, 36.80)	0.373
Heart rate, beats/min	91.00 (80.00, 105.00)	90.00 (80.00, 105.00)	93.50 (82.00, 107.00)	**0.043**	89.00 (81.00, 104.25)	88.00 (78.00, 105.00)	90.50 (82.00, 103.00)	0.424
Respiratory rate, breaths/min	20.00 (20.00, 22.00)	20.00 (20.00, 22.00)	21.00 (20.00, 23.00)	**7.288×10^{-6}**	20.00 (20.00, 22.00)	20.00 (20.00, 22.00)	21.00 (20.00, 22.00)	0.554
Systolic blood pressure, mmHg	127.50 (113.00, 141.25)	128.00 (114.00, 142.00)	125.00 (111.00, 140.00)	0.103	127.00 (111.00, 141.00)	127.00 (109.50, 140.00)	127.00 (112.75, 141.25)	0.498
Diastolic blood pressure, mmHg	75.00 (65.00, 84.00)	75.00 (65.00, 84.00)	73.00 (64.00, 83.00)	0.180	77.00 (65.00, 85.00)	76.50 (63.75, 85.25)	77.00 (66.00, 85.00)	0.732
**Underlying conditions**								
Hypertension	410 (38.25)	309 (41.31)	101 (31.17)	**0.002**	80 (34.48)	39 (33.62)	41 (35.34)	0.782
Coronary artery disease	201 (18.75)	132 (17.65)	69 (21.30)	0.160	36 (15.52)	17 (14.66)	19 (16.38)	0.717
Cerebrovascular disease	151 (14.09)	113 (15.11)	38 (11.73)	0.144	27 (11.64)	10 (8.62)	17 (14.66)	0.152
Diabetes	341 (31.81)	218 (29.14)	123 (37.96)	**0.004**	88 (37.93)	47 (40.52)	41 (35.34)	0.417
Chronic kidney disease	142 (13.25)	88 (11.76)	54 (16.67)	**0.030**	38 (16.38)	21 (18.10)	17 (14.66)	0.478

Variable	Before PSM				After PSM			
	Total (n = 1072)	Non-AmB (n = 748)	AmB (n = 324)	P	Total (n = 232)	Non-AmB (n = 116)	AmB (n = 116)	P
Chronic liver disease	129 (12.03)	83 (11.10)	46 (14.20)	0.152	37 (15.95)	19 (16.38)	18 (15.52)	0.858
Chronic respiratory disease	437 (40.76)	275 (36.76)	162 (50.00)	**5.128×10^{-5}**	93 (40.09)	42 (36.21)	51 (43.97)	0.228
Autoimmune disease	154 (14.37)	109 (14.57)	45 (13.89)	0.770	34 (14.66)	14 (12.07)	20 (17.24)	0.265
Malignancy	204 (19.03)	151 (20.19)	53 (16.36)	0.142	47 (20.26)	22 (18.97)	25 (21.55)	0.624
Organ transplantation	77 (7.18)	19 (2.54)	58 (17.90)	**3.715×10^{-19}**	29 (12.50)	15 (12.93)	14 (12.07)	0.843
**Treatment**								
Biologic therapy	167 (15.58)	49 (6.55)	118 (36.42)	**3.194×10^{-35}**	41 (17.67)	20 (17.24)	21 (18.10)	0.863
Glucocorticoids	597 (55.69)	345 (46.12)	252 (77.78)	**9.580×10^{-22}**	157 (67.67)	79 (68.10)	78 (67.24)	0.888
Antibiotics	866 (80.78)	559 (74.73)	307 (94.75)	**2.170×10^{-14}**	207 (89.22)	105 (90.52)	102 (87.93)	0.525
Antiviral drugs	284 (26.49)	147 (19.65)	137 (42.28)	**1.248×10^{-14}**	87 (37.50)	44 (37.93)	43 (37.07)	0.892
Anti-inflammatory drugs	323 (30.13)	187 (25.00)	136 (41.98)	**2.654×10^{-8}**	77 (33.19)	37 (31.90)	40 (34.48)	0.676
**Clinical outcomes**								
ICU admission	490 (45.71)	278 (37.17)	212 (65.43)	**1.443×10^{-17}**	112 (48.28)	57 (49.14)	55 (47.41)	0.793
Invasive mechanical ventilation	364 (33.96)	191 (25.53)	173 (53.40)	**9.084×10^{-19}**	89 (38.36)	43 (37.07)	46 (39.66)	0.685
Non-invasive ventilation	148 (13.81)	59 (7.89)	89 (27.47)	**1.403×10^{-17}**	41 (17.67)	19 (16.38)	22 (18.97)	0.606
Length of hospital stay, days	18.00 (11.00, 35.00)	15.00 (10.00, 23.25)	39.50 (19.75, 71.00)	**1.614×10^{-42}**	25.00 (14.00, 46.00)	22.00 (14.00, 41.00)	28.00 (15.00, 47.00)	0.155

Data are presented as n (%) for categorical variables and median (interquartile range) for continuous variables. The Mann–Whitney U test was used to compare continuous variables, and the χ^2 test or Fisher's exact test was used for categorical variables. $P < 0.05$ was considered statistically significant and is presented in bold.
Abbreviations: BMI, body mass index; ICU, intensive care unit.

Table S3. Detailed clinical characteristics of the after-PSM cohort

Variables	Total (n = 232)	Non-AmB (n = 116)	AmB (n = 116)	P
**Demographics**				
Age, years	63.00 (52.75, 70.00)	64.00 (51.75, 72.00)	63.00 (54.00, 69.00)	0.714
Male sex	160 (68.97)	79 (68.10)	81 (69.83)	0.777
BMI, kg/m^2	22.38 (19.59, 24.98)	22.89 (19.81, 25.13)	22.12 (19.46, 24.65)	0.243
**Vital signs**				
Temperature, °C	36.50 (36.20, 36.80)	36.50 (36.20, 36.80)	36.50 (36.30, 36.80)	0.373
Heart rate, beats/min	89.00 (81.00, 104.25)	88.00 (78.00, 105.00)	90.50 (82.00, 103.00)	0.424
Respiratory rate, breaths/min	20.00 (20.00, 22.00)	20.00 (20.00, 22.00)	21.00 (20.00, 22.00)	0.554
Systolic blood pressure, mmHg	127.00 (111.00, 141.00)	127.00 (109.50, 140.00)	127.00 (112.75, 141.25)	0.498
Diastolic blood pressure, mmHg	77.00 (65.00, 85.00)	76.50 (63.75, 85.25)	77.00 (66.00, 85.00)	0.732
**Underlying conditions**				
Hypertension	80 (34.48)	39 (33.62)	41 (35.34)	0.782
Coronary artery disease	36 (15.52)	17 (14.66)	19 (16.38)	0.717
Cerebrovascular disease	27 (11.64)	10 (8.62)	17 (14.66)	0.152
Diabetes	88 (37.93)	47 (40.52)	41 (35.34)	0.417
Chronic kidney disease	38 (16.38)	21 (18.10)	17 (14.66)	0.478
Chronic liver disease	37 (15.95)	19 (16.38)	18 (15.52)	0.858
Chronic respiratory disease	93 (40.09)	42 (36.21)	51 (43.97)	0.228
Autoimmune disease	34 (14.66)	14 (12.07)	20 (17.24)	0.265
Malignancy	47 (20.26)	22 (18.97)	25 (21.55)	0.624
Organ transplantation	29 (12.50)	15 (12.93)	14 (12.07)	0.843
**Laboratory tests at admission**				
Leukocyte count, 10^9/L	9.00 (6.35, 12.69)	8.98 (6.06, 12.30)	9.04 (6.54, 12.81)	0.456
Neutrophil count, 10^9/L	7.18 (4.37, 10.75)	7.20 (4.06, 10.66)	6.83 (4.44, 10.78)	0.676
Lymphocyte count, 10^9/L	1.02 (0.58, 1.64)	0.93 (0.52, 1.58)	1.10 (0.62, 1.69)	0.096
Platelet count, 10^9/L	193.00 (136.00, 277.00)	181.50 (119.75, 266.00)	210.00 (148.00, 287.00)	0.050
CRP, mg/L	30.18 (5.69, 105.65)	33.16 (7.06, 94.48)	27.73 (5.28, 116.53)	0.637
Procalcitonin, ng/mL	0.20 (0.20, 0.50)	0.20 (0.20, 0.72)	0.20 (0.11, 0.40)	0.184
Serum galactomannan index	0.37 (0.17, 2.79)	0.30 (0.17, 1.06)	0.54 (0.18, 3.15)	0.188
Urea, mmol/L	6.67 (4.60, 10.84)	7.35 (4.62, 12.48)	6.37 (4.42, 9.95)	0.090
ALT, U/L	23.00 (14.75, 45.50)	22.00 (15.00, 50.00)	25.00 (14.00, 43.00)	0.473
AST, U/L	25.00 (17.00, 43.25)	25.00 (16.00, 50.50)	25.50 (17.00, 41.25)	0.830

Variables	Total (n = 232)	Non-AmB (n = 116)	AmB (n = 116)	P
**Treatment**				
Biologic therapy	41 (17.67)	20 (17.24)	21 (18.10)	0.863
Glucocorticoids	157 (67.67)	79 (68.10)	78 (67.24)	0.888
Antibiotics	207 (89.22)	105 (90.52)	102 (87.93)	0.525
Antiviral drugs	87 (37.50)	44 (37.93)	43 (37.07)	0.892
Anti-inflammatory drugs	77 (33.19)	37 (31.90)	40 (34.48)	0.676
**Clinical outcomes**				
In-hospital mortality	26 (11.21)	13 (11.21)	13 (11.21)	1.000
ICU admission	112 (48.28)	57 (49.14)	55 (47.41)	0.793
Invasive mechanical ventilation	89 (38.36)	43 (37.07)	46 (39.66)	0.685
Non-invasive ventilation	41 (17.67)	19 (16.38)	22 (18.97)	0.606
Length of hospital stay, days	25.00 (14.00, 46.00)	22.00 (14.00, 41.00)	28.00 (15.00, 47.00)	0.155
Viral infection after antifungal therapy	34 (14.66)	9 (7.76)	25 (21.55)	**0.003**

Data are presented as n (%) for categorical variables and median (interquartile range) for continuous variables. The Mann–Whitney U test was used to compare continuous variables, and the χ^2 test or Fisher's exact test was used for categorical variables. $P < 0.05$ was considered statistically significant and is presented in bold.

Abbreviations: ALT, alanine aminotransferase; AmB, amphotericin B; AST, aspartate aminotransferase; BMI, body mass index; CRP, C-reactive protein; ICU, intensive care unit; PSM, propensity score matching.

Table S4. Multivariable logistic regression of factors associated with incident viral infection after antifungal therapy in the overall cohort

Variable	Univariate		Multivariate	
	P	OR (95%CI)	P	OR (95%CI)
Age ≥60 years	**0.014**	0.61 (0.41–0.91)	0.128	0.72 (0.48–1.10)
Male sex	0.499	1.16 (0.76–1.77)		
Hypertension	0.061	1.46 (0.98–2.16)		
Coronary artery disease	1.000	1.00 (0.61–1.65)		
Cerebrovascular disease	0.524	1.19 (0.70–2.04)		
Diabetes	0.250	1.27 (0.84–1.91)		
Chronic kidney disease	0.130	1.49 (0.89–2.51)		
Chronic liver disease	0.883	0.96 (0.52–1.76)		
Chronic respiratory disease	0.589	0.90 (0.60–1.34)		
Autoimmune disease	0.587	1.16 (0.68–1.98)		
Malignancy	0.179	0.68 (0.39–1.19)		
Organ transplantation	0.256	1.48 (0.75–2.88)		
Biologic therapy	**2.347×10^{-7}**	3.15 (2.04–4.87)	0.092	1.54 (0.93–2.56)
Glucocorticoids	**5.027×10^{-4}**	2.14 (1.39–3.29)	0.295	1.30 (0.80–2.11)
AmB	**7.109×10^{-13}**	4.44 (2.95–6.66)	**7.174×10^{-8}**	3.45 (2.20–5.41)

Data are presented as odds ratios (OR) with 95% confidence intervals (CI). Univariable and multivariable logistic regression models were used for analysis, and all statistical tests were two-sided. Variables with $P < 0.05$ in the univariable analysis were included in the multivariable model using a forward stepwise selection procedure. $P < 0.05$ was considered statistically significant and is presented in bold.

Abbreviations: AmB, amphotericin B; PSM, propensity-score matching.

Table S5. Sequences of siRNAs targeting *GBA1*

siRNA		Sequence (5′ to 3′)
GBA1-siRNA1	Sense	5′- CACAUACUGUGACUCCUUUTT -3′
	Antisense	5′- AAAGGAGUCACAGUAUGUGTT -3′
GBA1-siRNA2	Sense	5′- CAGAACAGAAGUUCCAGAATT -3′
	Antisense	5′- UUCUGGAACUUCUGUUCUGTT -3′
GBA1-siRNA3	Sense	5′- CUACUUAAAUGGUACUUCUTT -3′
	Antisense	5′- AGAAGUACGAUUUAAGUAGTT -3′
GBA1-siRNA4	Sense	5′- GCGUAACUUUGUCGACAGUTT -3′
	Antisense	5′- ACUGUCGACAAAGUUACGCTT -3′
NC-siRNA	Sense	5′- UUCUCCGAACGUGUCACGUTT -3′
	Antisense	5′- ACGUGACACGUUCGGAGAATT -3′

Table S6. Primer sequences for qRT-PCR

	Forward primer (5′ to 3′)	Reverse primer (5′ to 3′)
influenza A virus (H1N1) strain A/PR/8/34 *nucleoprotein (NP)*	CAGCCTAATCAGACCAAATG	TACCTGCTTCTCAGTTCAAG
Mouse *Gapdh*	TGCCCCCATGTTTGTGATG	TGTGGTCATGAGCCCTTCC
Human / *Chlorocebus GAPDH*	CTGCACCACCAACTGCTTAG	GAGCTTCCCGTTCAGCTCAG
Human *IFITM3*	CTGGGCTTCATAGCATTCGC	CCATAGGCCTGGAAGATCAGC
Chlorocebus IFITM3	CATAGCGTTCGCCTACTCCG	GGCGAGGAATGGAAGGTGG
Human *GBA1*	CTCAAGACCAATGGAGCGGT	TGATGTTCAGGGGTGAAGCC
