# Supplementary material for: Amphotericin B promotes respiratory viral entry by enhancing late endosomal maturation and fusion via glucocerebrosidase-mediated ceramide remodeling
Source: Nat Commun. 2026 Mar 9;17:3670. doi: 10.1038/s41467-026-70095-x (PMC13100133; doi:10.1038/s41467-026-70095-x)
Supplement: Supplementary file 2 — Description Of Additional Supplementary File [file 41467_2026_70095_MOESM2_ESM.pdf]

Description of additional supplementary files

Supplementary Data 1:

Quantitative targeted lipidomics dataset of late endosomes from A549 cells
