# Supplementary material for: Amphotericin B promotes respiratory viral entry by enhancing late endosomal maturation and fusion via glucocerebrosidase-mediated ceramide remodeling
Source: Nat Commun. 2026 Mar 9;17:3670. doi: 10.1038/s41467-026-70095-x (PMC13100133; doi:10.1038/s41467-026-70095-x)
Supplement: Supplementary file 4 — Reporting Summary [file 41467_2026_70095_MOESM4_ESM.pdf]

nature portfolio

Corresponding author(s): Bin Cao, Zai Wang, Peigang Wang, and Min Luo

Last updated by author(s): Feb 11, 2026

Reporting Summary

Nature Portfolio wishes to improve the reproducibility of the work that we publish. This form provides structure for consistency and transparency in reporting. For further information on Nature Portfolio policies, see our Editorial Policies and the Editorial Policy Checklist.

Statistics

For all statistical analyses, confirm that the following items are present in the figure legend, table legend, main text, or Methods section.

n/a	Confirmed	
☐	☒	The exact sample size (*n*) for each experimental group/condition, given as a discrete number and unit of measurement
☐	☒	A statement on whether measurements were taken from distinct samples or whether the same sample was measured repeatedly
☐	☒	The statistical test(s) used AND whether they are one- or two-sided<br>*Only common tests should be described solely by name; describe more complex techniques in the Methods section.*
☐	☒	A description of all covariates tested
☐	☒	A description of any assumptions or corrections, such as tests of normality and adjustment for multiple comparisons
☐	☒	A full description of the statistical parameters including central tendency (e.g. means) or other basic estimates (e.g. regression coefficient) AND variation (e.g. standard deviation) or associated estimates of uncertainty (e.g. confidence intervals)
☐	☒	For null hypothesis testing, the test statistic (e.g. *F*, *t*, *r*) with confidence intervals, effect sizes, degrees of freedom and *P* value noted<br>*Give P values as exact values whenever suitable.*
☒	☐	For Bayesian analysis, information on the choice of priors and Markov chain Monte Carlo settings
☒	☐	For hierarchical and complex designs, identification of the appropriate level for tests and full reporting of outcomes
☒	☐	Estimates of effect sizes (e.g. Cohen's *d*, Pearson's *r*), indicating how they were calculated

Our web collection on statistics for biologists contains articles on many of the points above.

Software and code

Policy information about availability of computer code

Data collection

For data collection, the following instruments and associated software were used:
1. Western Blot Imaging: ChemiDoc™ Gel Imaging System (Bio-Rad, USA) with Image Lab Software version 6.0 (Bio-Rad, USA).
2. Immunofluorescence Imaging: LSM 800 Confocal Laser Scanning Microscope (Zeiss, Germany) running ZEN 3.4 Fine Software (Zeiss, Germany).
3. Immunohistochemistry and Histological Scanning: Pannoramic MIDI II Digital Scanner (3DHISTECH, Hungary) with SlideViewer Software version 2.6.0 (3DHISTECH, Hungary).
4. Reverse Transcription Quantitative PCR: QuantStudio™ 5 Real-Time PCR System (Thermo Fisher Scientific, USA) using QuantStudio™ Design & Analysis Software version 1.5.1 (Thermo Fisher Scientific, USA).
5. Absorbance, Fluorescence, and Luminescence Assays: Spark® multimode microplate reader (Tecan, Switzerland) controlled by SparkControl™ Software version 2.2 (Tecan, Switzerland).
6. Flow Cytometry: CytoFLEX LX Flow Cytometer (Beckman Coulter, USA) with CytExpert Software version 2.5.0.77 (Beckman Coulter, USA).
7. Surface Plasmon Resonance: Biacore T200 System (Cytiva, USA) operated using Biacore T200 Evaluation Software version 4.0 (Cytiva, USA).
8. Lipidomics LC-MS: ExionLC-AD liquid chromatography system coupled with a Sciex QTRAP 6500 PLUS mass spectrometer (Sciex, USA).
9. Shotgun Proteomic Identification: Q Exactive HF-X mass spectrometer (Thermo Fisher Scientific, USA) coupled with an Easy nLC 1200 system (Thermo Fisher Scientific, USA) using MaxQuant software version 1.6.1.0 (Max Planck Institute of Biochemistry, Germany).
10. DIA-based Quantitative Proteomic Analysis: Orbitrap Astral mass spectrometer (Thermo Fisher Scientific, USA) coupled with a Vanquish Neo UHPLC system (Thermo Fisher Scientific, USA) using DIA-NN software version 1.8.1 (Dense Mass, Germany).

Data analysis

For data analysis, the following software packages were used:
1. Western blot, immunofluorescence, and immunohistochemistry quantification: ImageJ software version 1.53k (National Institutes of

Health, USA).
2. Statistical analyses and graph plotting: GraphPad Prism version 9.5.1 (GraphPad Software, USA) and R 4.3.3 (Austria).

For manuscripts utilizing custom algorithms or software that are central to the research but not yet described in published literature, software must be made available to editors and reviewers. We strongly encourage code deposition in a community repository (e.g. GitHub). See the Nature Portfolio guidelines for submitting code & software for further information.

Data

Policy information about availability of data

All manuscripts must include a data availability statement. This statement should provide the following information, where applicable:

- Accession codes, unique identifiers, or web links for publicly available datasets
- A description of any restrictions on data availability
- For clinical datasets or third party data, please ensure that the statement adheres to our policy

The mass spectrometry proteomics data generated in this study have been deposited in the ProteomeXchange Consortium under accession codes PXD064601 [https://www.ebi.ac.uk/pride/archive/projects/PXD064601] and PXD064637 [https://www.ebi.ac.uk/pride/archive/projects/PXD064637]. The targeted lipidomics data are provided in the Supplementary Data. Raw lipidomics instrument files are unavailable due to third-party intellectual property restrictions associated with the service provider (LipidALL Technologies), but the provided quantitative dataset is sufficient to interpret and validate the reported findings. Patient clinical data are not publicly available to strictly comply with data privacy laws and ethical regulations protecting patient confidentiality. However, de-identified clinical data will be made available to researchers upon reasonable request. Access requests should be submitted to the corresponding author and must include a research proposal and a signed Data Use Agreement. The corresponding author will review requests in accordance with the ethics committee's requirements and respond within 4 weeks. All other data supporting the findings of this study are available within the article, its Supplementary Information, or the Source Data file. Source data are provided with this paper.

Research involving human participants, their data, or biological material

Policy information about studies with human participants or human data. See also policy information about sex, gender (identity/presentation), and sexual orientation and race, ethnicity and racism.

Reporting on sex and gender	In the retrospective study, we included 1072 patients. Of these, 356 were identified as female and 716 as male based on medical records indicating biological sex. No data on gender identity were collected. Our primary focus was on the prognostic impact of systemic amphotericin B treatment, rather than on differences based on sex or gender. Consequently, we did not conduct statistical analyses or stratify outcomes by sex or gender.
Reporting on race, ethnicity, or other socially relevant groupings	n/a
Population characteristics	Patient characteristics are summarized in Supplementary Tables S2 and S3, covering demographics, vital signs, underlying conditions, admission laboratory tests, treatment, and clinical outcomes.
Recruitment	We conducted a retrospective cohort study using the electronic medical records of adult inpatients at China-Japan Friendship Hospital between September 2016 and September 2025. Patients were included if they had a positive Aspergillus culture from a respiratory sample and received systemic antifungal therapy. Patients with insufficient medical records were excluded. Based on treatment, patients were classified into two groups: the AmB group was defined by the administration of systemic AmB for ≥3 consecutive days, and the Non-AmB group by the absence of such treatment.
Ethics oversight	The retrospective clinical cohort study was performed in line with the principles of the Ethics Committee of China-Japan Friendship Hospital (Approval number 2022KY-052) in accordance with the Declaration of Helsinki. The requirement for informed consent was waived by the Ethics Committee due to the retrospective nature of the study, and no participant compensation was provided.

Note that full information on the approval of the study protocol must also be provided in the manuscript.

Field-specific reporting

Please select the one below that is the best fit for your research. If you are not sure, read the appropriate sections before making your selection.

☒ Life sciences ☐ Behavioural & social sciences ☐ Ecological, evolutionary & environmental sciences

For a reference copy of the document with all sections, see nature.com/documents/nr-reporting-summary-flat.pdf

Life sciences study design

All studies must disclose on these points even when the disclosure is negative.

Sample size	Sample sizes for each experiment are indicated in the figures or corresponding figure legends. No statistical methods were used to predetermine sample sizes; instead, they were determined based on prior experience and published literature.
Data exclusions	No data were excluded.

Replication	All attempts at replication were successful. The number of independent biological replicates for each experiment is clearly indicated in the corresponding figure legends.
Randomization	Groups were randomized before infection or treatment.
Blinding	Investigators were not blinded to group allocation during the experiments or outcome assessments due to the study's design. Although blinding was not feasible, all measurements and quantifications were conducted using standardized, objective protocols to minimize bias.

Reporting for specific materials, systems and methods

We require information from authors about some types of materials, experimental systems and methods used in many studies. Here, indicate whether each material, system or method listed is relevant to your study. If you are not sure if a list item applies to your research, read the appropriate section before selecting a response.

Materials & experimental systems

n/a	Involved in the study	
☐	☒	Antibodies
☐	☒	Eukaryotic cell lines
☒	☐	Palaeontology and archaeology
☐	☒	Animals and other organisms
☐	☒	Clinical data
☒	☐	Dual use research of concern
☒	☐	Plants

Methods

n/a	Involved in the study	
☒	☐	ChIP-seq
☐	☒	Flow cytometry
☒	☐	MRI-based neuroimaging

Antibodies

Antibodies used

Primary Antibodies (all commercially available):
Rabbit anti-SARS-CoV-2 nucleocapsid protein (Sino Biological; catalog no. 40143-R001; dilution 1:500 for immunohistochemistry)
Mouse anti-influenza A virus nucleoprotein (Abcam; catalog no. ab20343; dilution 1:2,000 for Western blot, 1:100 for immunofluorescence)
Mouse anti-β-actin (Sigma-Aldrich; catalog no. A5316; dilution 1:1,000 for Western blot)
Mouse anti-Lamin A/C (Cell Signaling Technology; catalog no. 4777S; dilution 1:2,000 for Western blot)
Rabbit anti-RAB5A (Cell Signaling Technology; catalog no. 2143; dilution 1:1,000 for Western blot)
Rabbit anti-RAB11 (Proteintech; catalog no. 15903-1-AP; dilution 1:1,000 for Western blot)
Rabbit anti-RAB7 (Abcam; catalog no. ab137029; dilution 1:1,000 for Western blot, 1:100 for immunofluorescence)
Rabbit anti-LAMP1 (Cell Signaling Technology; catalog no. 9091S; dilution 1:1,000 for Western blot)
Rabbit anti-LAMP2 (Thermo Fisher Scientific; catalog no. PA1-655; dilution 1:1,000 for Western blot)
Rabbit anti-NPC1 (Abcam; catalog no. ab134113; dilution 1:2,000 for Western blot)
Rabbit anti-GCase (Abcam; catalog no. ab128879; dilution 1:1,000 for Western blot)
Rabbit anti-EEA1 (Cell Signaling Technology; catalog no. 3288; dilution 1:100 for immunofluorescence)
Biotinylated Sambucus nigra lectin (Vector; catalog no. B-1305-2; dilution 1:200 for immunofluorescence)

Secondary Antibodies (all commercially available):
HRP-conjugated anti-rabbit IgG (Solarbio; catalog no. SE134; dilution 1:5,000 for Western blot, 1:200 for immunohistochemistry)
HRP-conjugated anti-mouse IgG (Solarbio; catalog no. SE131; dilution 1:5,000 for Western blot)
Alexa Fluor 488-conjugated donkey anti-mouse IgG (Thermo Fisher Scientific; catalog no. A32766; dilution 1:1,000 for immunofluorescence)
Alexa Fluor 555-conjugated donkey anti-mouse IgG (Thermo Fisher Scientific; catalog no. A31570; dilution 1:1,000 for immunofluorescence)
Alexa Fluor 555-conjugated donkey anti-rabbit IgG (Thermo Fisher Scientific; catalog no. A32794; dilution 1:1,000 for immunofluorescence)
Alexa Fluor 488-conjugated Streptavidin (BioLegend; catalog no. 405235; dilution 1:400 for immunofluorescence)

Validation

Primary Antibodies (all commercially available):
Rabbit anti-SARS-CoV-2 nucleocapsid protein (Sino Biological; catalog no. 40143-R001; Reactivity and QC at: RRID: AB_2827974) (https://cdn1.sinobiological.com/reagent/40143-R001.pdf)
Mouse anti-influenza A virus nucleoprotein (Abcam; catalog no. ab20343; Reactivity and QC at: RRID: AB_445525) (https://doc.abcam.com/datasheets/active/ab20343/en-us/influenza-a-virus-nucleoprotein-antibody-aa5h-ab20343.pdf)
Mouse anti-β-actin (Sigma-Aldrich; catalog no. A5316; Reactivity and QC at: RRID: AB_476743) (https://www.sigmaaldrich.com/specification-sheets/226/889/A5316-BULK________SIGMA____.pdf)
Mouse anti-Lamin A/C (Cell Signaling Technology; catalog no. 4777S; Reactivity and QC at: RRID: AB_10545756) (https://www.cellsignal.com/products/4777/datasheet?images=1&protocol=0)
Rabbit anti-RAB5A (Cell Signaling Technology; catalog no. 2143; Reactivity and QC at: RRID: AB_823625) (https://www.cellsignal.com/products/2143/datasheet?images=1&protocol=0)
Rabbit anti-RAB11 (Proteintech; catalog no. 15903-1-AP; Reactivity and QC at: RRID: AB_2173458) (https://www.ptglab.com/products/pictures/pdf/15903-1-AP.pdf)
Rabbit anti-RAB7 (Abcam; catalog no. ab137029; Reactivity and QC at: RRID: AB_2629474) (https://doc.abcam.com/datasheets/active/ab137029/en-us/rab7-antibody-epr7589-late-endosome-marker-ab137029.pdf)

Rabbit anti-LAMP1 (Cell Signaling Technology; catalog no. 9091S; Reactivity and QC at: RRID: AB_2687579) (https://www.cellsignal.com/products/9091/datasheet?images=1&protocol=0)
Rabbit anti-LAMP2 (Thermo Fisher Scientific; catalog no. PA1-655; Reactivity and QC at: RRID: AB_2134625) (https://www.thermofisher.com/order/genome-database/dataSheetPdf?producttype=antibody&productsubtype=antibody_primary&productId=PA1-655&version=Local)
Rabbit anti-NPC1 (Abcam; catalog no. ab134113; Reactivity and QC at: RRID: AB_2734695) (https://doc.abcam.com/datasheets/active/ab134113/en-us/niemann-pick-c1-antibody-epr5209-ab134113.pdf)
Rabbit anti-GCase (Abcam; catalog no. ab128879; Reactivity and QC at: RRID: AB_11144121) (https://doc.abcam.com/datasheets/active/ab128879/en-us/gba-antibody-epr51433-ab128879.pdf)
Rabbit anti-EEA1 (Cell Signaling Technology; catalog no. 3288; Reactivity and QC at: RRID: AB_2096811) (https://www.cellsignal.com/products/3288/datasheet?images=1&protocol=0)
Biotinylated Sambucus nigra lectin (Vector; catalog no. B-1305-2; Reactivity and QC at: RRID: AB_2336718) (https://vectorlabs.com/products/biotinylated-sambucus-nigra-lectin-sna-ebl/?print-products=pdf&variation=&)

Secondary Antibodies (all commercially available):
HRP-conjugated anti-rabbit IgG (Solarbio; catalog no. SE134; Reactivity and QC at: RRID: AB_2797593) (https://en.solarbio.com/goodsInfo?id=1546)
HRP-conjugated anti-mouse IgG (Solarbio; catalog no. SE131; Reactivity and QC at: RRID: AB_2797595) (https://en.solarbio.com/goodsInfo?id=1547)
Alexa Fluor 488-conjugated donkey anti-mouse IgG (Thermo Fisher Scientific; catalog no. A32766; Reactivity and QC at: RRID: AB_2762823) (https://www.thermofisher.cn/order/genome-database/dataSheetPdf?producttype=antibody&productsubtype=antibody_secondary&productId=A32766&version=Local)
Alexa Fluor 555-conjugated donkey anti-mouse IgG (Thermo Fisher Scientific; catalog no. A31570; Reactivity and QC at: RRID: AB_2536180) (https://www.thermofisher.cn/order/genome-database/dataSheetPdf?producttype=antibody&productsubtype=antibody_secondary&productId=A-31570&version=Local)
Alexa Fluor 555-conjugated donkey anti-rabbit IgG (Thermo Fisher Scientific; catalog no. A32794; Reactivity and QC at: RRID: AB_2762834) (https://www.thermofisher.cn/order/genome-database/dataSheetPdf?producttype=antibody&productsubtype=antibody_secondary&productId=A32794&version=Local)
Alexa Fluor 488-conjugated Streptavidin (BioLegend; catalog no. 405235) (https://www.biolegend.com/nb-no/products/alexa-fluor-488-streptavidin-9304?pdf=true&displayInline=true&leftRightMargin=15&topBottomMargin=15&filename=Alexa%20Fluor®%20488%20Streptavidin.pdf&v=20251213043807)

Eukaryotic cell lines

Policy information about cell lines and Sex and Gender in Research

Cell line source(s)	The cell lines used in this study included A549 (Cat# CCL-185), HULEC-5a (Cat# CRL-3244), 293T (Cat# CRL-3216), COS-7 (Cat# CRL-1651), HeLa (Cat# CCL-2), MDCK (Cat# CCL-34), and Vero E6 (Cat# CRL-1586) cells, all obtained from the American Type Culture Collection (ATCC, USA). Huh7 cells were kindly provided by Dr. Xuanling Shi (Tsinghua University). A CRISPR-Cas9-mediated GBA1 knockout 293T cell line (Cat# SY-KO-00256) was acquired from Cyagen Biosciences (China). Stable hACE2-expressing cell lines (HeLa-hACE2 and A549-hACE2 cells) were generated in-house via retroviral transduction.
Authentication	Eukaryotic cell lines were obtained from ATCC, other reputable vendors, or as generous gifts from collaborating laboratories. All cell lines were authenticated by the original suppliers or through STR profiling. Upon receipt, morphology was confirmed by light microscopy, and all lines exhibited the expected growth and behavior.
Mycoplasma contamination	All cell lines were routinely screened for mycoplasma contamination and were confirmed to be negative.
Commonly misidentified lines (See ICLAC register)	No commonly misidentified cell lines were used in this study.

Animals and other research organisms

Policy information about studies involving animals; ARRIVE guidelines recommended for reporting animal research, and Sex and Gender in Research

Laboratory animals	SPF 6–8-week-old male C57BL/6J mice and LVG Golden Syrian hamsters were obtained from Beijing Vital River Laboratory Animal Technology Co., Ltd. (China). The animals were housed five per cage with ad libitum access to food and water at a temperature of 24 ± 2 °C, relative humidity of 50 ± 5%, and a 12-h light/12-h dark cycle. They were acclimated to the facility for 5 days prior to the experiments. The number of animals used for each experiment (n) is reported in the figure legends. Sex was not included as a biological variable as all experiments were performed using male animals to reduce variability and to maintain consistency across cohorts.
Wild animals	No wild animals were involved in the study.
Reporting on sex	The experiments exclusively involved male animals. The findings are not reported by sex, and no sex-based analysis was performed.
Field-collected samples	This research did not utilize any field-collected samples.
Ethics oversight	All animal experiments were conducted in strict accordance with the Guide for the Care and Use of Medical Laboratory Animals (Ministry of Health, People's Republic of China) and received approval from two independent ethics committees. Specifically, the protocols for the murine experiments were evaluated and approved by the Animal Ethics Committee of the Institute of Biophysics, Chinese Academy of Sciences (Approval number ABSL-2-2023019), whereas the procedures for hamster experiments were reviewed

and sanctioned by the Institutional Animal Care and Use Committee of the Institute of Laboratory Animal Science, Peking Union Medical College & Chinese Academy of Medical Sciences (Approval number XZG23001).

Note that full information on the approval of the study protocol must also be provided in the manuscript.

Clinical data

Policy information about clinical studies

All manuscripts should comply with the ICMJE guidelines for publication of clinical research and a completed CONSORT checklist must be included with all submissions.

Clinical trial registration	n/a
Study protocol	n/a
Data collection	Data were retrospectively extracted from the electronic medical record system. Collected variables encompassed demographics, vital signs, underlying comorbidities, laboratory and microbiological results, detailed records of antifungal and concomitant medications, and clinical outcomes.
Outcomes	The primary outcome was defined as the occurrence of laboratory-confirmed respiratory viral infection following the initiation of antifungal therapy.

Plants

Seed stocks	*Report on the source of all seed stocks or other plant material used. If applicable, state the seed stock centre and catalogue number. If plant specimens were collected from the field, describe the collection location, date and sampling procedures.*
Novel plant genotypes	*Describe the methods by which all novel plant genotypes were produced. This includes those generated by transgenic approaches, gene editing, chemical/radiation-based mutagenesis and hybridization. For transgenic lines, describe the transformation method, the number of independent lines analyzed and the generation upon which experiments were performed. For gene-edited lines, describe the editor used, the endogenous sequence targeted for editing, the targeting guide RNA sequence (if applicable) and how the editor was applied.*
Authentication	*Describe any authentication procedures for each seed stock used or novel genotype generated. Describe any experiments used to assess the effect of a mutation and, where applicable, how potential secondary effects (e.g. second site T-DNA insertions, mosiacism, off-target gene editing) were examined.*

Flow Cytometry

Plots

Confirm that:

☒ The axis labels state the marker and fluorochrome used (e.g. CD4-FITC).

☒ The axis scales are clearly visible. Include numbers along axes only for bottom left plot of group (a 'group' is an analysis of identical markers).

☒ All plots are contour plots with outliers or pseudocolor plots.

☒ A numerical value for number of cells or percentage (with statistics) is provided.

Methodology

Sample preparation	A549 cells were treated as follows to assess specific endocytic pathways:<br>Clathrin-mediated endocytosis: Incubation with 25 µg/mL Alexa Fluor 488-conjugated transferrin (TF-488) at 37 °C for 5 min.<br>Macropinocytosis: Incubation with 1 mg/mL tetramethylrhodamine-labeled 70-kDa dextran (TMR-Dextran) at 37 °C for 40 min.<br>Caveolin-dependent uptake: Binding of 10 µg/mL Alexa Fluor 555-conjugated cholera toxin subunit B (CTB-555) on ice for 10 min, followed by internalization at 37 °C for 30 min.<br>Following the respective treatments and appropriate washes to remove non-internalized probes, all samples were processed uniformly: cells were trypsinized and pelleted. To exclude dead cells, samples were stained with the LIVE/DEAD™ Fixable Near-IR (876) Dead Cell Stain Kit according to the manufacturer's instructions. Finally, cells were resuspended in PBS containing 2% FBS for flow cytometric analysis.
Instrument	CytoFLEX LX Flow Cytometer (Beckman Coulter, USA)
Software	CytExpert Software version 2.5.0.77 (Beckman Coulter, USA)
Cell population abundance	10,000 cells per tube were collected.
Gating strategy	Cells were first gated based on forward scatter (FSC-A) and side scatter (SSC-A) parameters to identify the cell population. Single cells were then selected through doublet exclusion using FSC-A and FSC-H. Dead cells were subsequently excluded

based on Live/Dead Near-IR staining. Finally, positive gates for internalized TF-488, TMR-Dextran, or CTB-555 were established using untreated or unstained control samples to ensure accurate quantification of endocytic uptake.

☒ Tick this box to confirm that a figure exemplifying the gating strategy is provided in the Supplementary Information.
